# Electrical magnetochiral effect induced by chiral spin fluctuations

**DOI:** 10.1038/s41467-017-01094-2

**Published:** 2017-10-11

**Authors:** T. Yokouchi, N. Kanazawa, A. Kikkawa, D. Morikawa, K. Shibata, T. Arima, Y. Taguchi, F. Kagawa, Y. Tokura

**Affiliations:** 1 Department of Applied Physics, The University of Tokyo, Tokyo, 113-8656 Japan; 2 RIKEN Center for Emergent Matter Science (CEMS), Wako, 351-0198 Japan; 3 Department of Advanced Materials Science, The University of Tokyo, Kashiwa, 277-8561 Japan

## Abstract

Chirality of matter can produce unique responses in optics, electricity and magnetism. In particular, magnetic crystals transmit their handedness to the magnetism via antisymmetric exchange interaction of relativistic origin, producing helical spin orders as well as their fluctuations. Here we report for a chiral magnet MnSi that chiral spin fluctuations manifest themselves in the electrical magnetochiral effect, i.e. the nonreciprocal and nonlinear response characterized by the electrical resistance depending on inner product of current and magnetic field. Prominent electrical magnetochiral signals emerge at specific temperature-magnetic field-pressure regions: in the paramagnetic phase just above the helical ordering temperature and in the partially-ordered topological spin state at low temperatures and high pressures, where thermal and quantum spin fluctuations are conspicuous in proximity of classical and quantum phase transitions, respectively. The finding of the asymmetric electron scattering by chiral spin fluctuations may explore new electromagnetic functionality in chiral magnets.

## Abstract (web summary)

The magnetism-induced chirality in electron transportation is of fundamental importantance in condensed matter physics but the origin is still unclear. Here the authors demonstrate that the asymmetric electron scattering by chiral spin fluctuations can be the key to the electrical magnetochiral effect in MnSi.

## Introduction

Transport phenomena related to magnetism, including spin fluctuations and chiral magnetism, provide rich physics and functionalities. For example, antiferromagnetic spin fluctuations are involved in formation of Cooper pairs in the high-temperature superconducting cuprates^1^, and quantum spin fluctuations break down the Fermi-liquid behaviour^2^. As for the chiral magnetism, real-space Berry phase related to non-coplanar spin textures with finite scalar spin chirality ${\chi _{ijk}} = {{\bf{S}}_i} \cdot \left( {{{\bf{S}}_j} \times {{\bf{S}}_k}} \right)$, where **S**_*n*_ (*n* = *i*, *j*, *k*) are adjacent three spins, can produce emergent magnetic field and hence the topological Hall effect^3, 4^. Despite appreciation of these two concepts, cooperative phenomena from spin fluctuations and spin chirality (**C**_*ij*_ = **S**_*i*_ × **S**_*j*_) have not fully been explored in charge transport phenomena. For their exploration, we focus on directional nonlinear magnetotransport with the resistance proportional to inner product of magnetic field (**B**) and current, termed electrical magnetochiral effect (eMChE)^5–7^. The eMChE is one kind of directional magnetotransport phenomena being odd against *B*, which are generally allowed in noncentrosymmetric systems. Recently, from the viewpoint of not only fundamental physics but also applications, such directional nonlinear transports are investigated, for example, in polar systems such as at interfaces between ferromagnetic metals and nonmagnetic heavy metals^8, 9^, at surfaces of magnetic and nonmagnetic topological insulator heterostructures^10^, and in polar bulk semiconductor^11^. As for chiral system, however, eMChE in chiral magnet has not been explored, and the relationship between eMChE and chiral magnetism remains elusive.

**Fig. 1:**
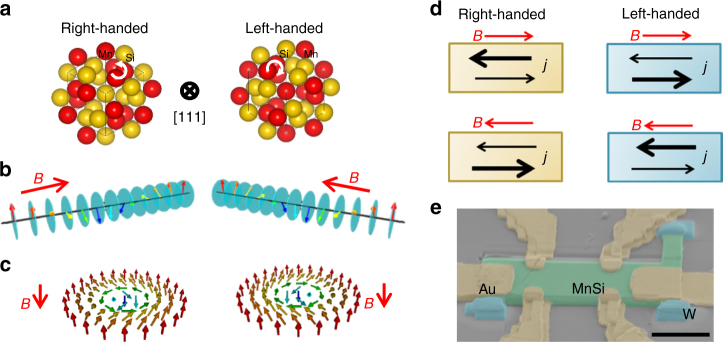
Crystal and spin structures of MnSi, and experimental configurations. **a**–**c** Crystal structures of right- and left-handed MnSi viewed from the [111] direction **a** and the corresponding spin structures of helical orders **b** and skyrmions **c**. We define the right- and left-handed MnSi as the atomic coordinates (*u*, *u*, *u*), (1/2 + *u*, 1/2 − *u*, 1/2 − *u*), (1/2 − *u*, −*u*, 1/2 + *u*), (−*u*, 1/2 + *u*, 1/2 − *u*) with *u*_Mn_ = 0.863, *u*_Si_ = 0.155 and with *u*_Mn_ = 0.137, *u*_Si_ = 0.845, respectively. **d** Experimental configurations for measurements of electrical magnetochiral effect and expected dichroic properties of current density. The bold arrows schematically represent paths with the larger current density at a constant electric field along the arrow direction. **e** A scanning electron microscope image of a MnSi thin plate sample: MnSi crystal (green), gold electrodes (yellow), tungsten for fixing the sample (light blue), and silicon stage (grey). *Scale bar*, 5 μm

Spin structures and their dynamics in chiral-lattice magnets bear chiral nature due to antisymmetric exchange interactions, such as Dzyaloshinskii-Moriya (DM) interaction (**D** · **C**_*ij*_); the sign of the DM vector **D** is intrinsically dependent on the crystalline chirality. As a consequence, the sign of their magnetic chirality, as defined for example by **r**_*ij*_ · **C**_*ij*_ (**r**_*ij*_ being the vector connecting *i*-th and *j*-th sites), is macroscopically coherent throughout the crystal, which can make the chirality dependent transport signals macroscopically visible. MnSi of the present focus has the noncentrosymmetric lattice structure, which can exist in two enantiomeric forms: right- and left-handed structures as shown in Fig. 1a. Due to the competition between the ferromagnetic exchange interaction and the DM interaction, there emerge various spin winding structures, whose modulation directions, *i*.*e*. magnetic helicity, are determined by handedness of the corresponding lattice structures. Below the magnetic ordering temperature *T*_c_ = 29.5 K, the long-period (~18 nm) helical spin structure (Fig. 1b) forms^12^. In addition, topological spin objects, skyrmions (Fig. 1c), condense in triangular-lattice (skyrmion-lattice state) at 0.1 T ≲ *B* ≲ 0.3 T just below *T*_c_^13^. Above *T*_c_, where the long-range magnetic orders disappear, short-range spin correlations still survive without losing the chiral nature^14–17^, as described as $\langle {{{( {\langle {{{\bf{C}}_{ij}}} \rangle - {{{\bf{C}}_{ij}}} } )}^2}} \rangle$. Strong enhancement of the chiral spin fluctuations around *T*_c_ has been theoretically proposed^18^ and demonstrated by polarized neutron scattering experiments^14–17^.

In the following we demonstrate that thermal and quantum spin fluctuations endowed with finite vector spin chirality, i.e. chiral spin fluctuations, produce an eMChE. We find that prominent electrical magnetochiral signals emerge at specific temperature-magnetic field-pressure regions: in the paramagnetic phase just above the helical ordering temperature and in the partially-ordered topological spin state at low temperatures and high pressures, where thermal and quantum spin fluctuations are conspicuous in proximity of classical and quantum phase transitions, respectively.

## Results

### Experimental design for detection of eMChE in MnSi

We found that the chiral spin fluctuations play a key role in the eMChE in MnSi. From the viewpoint of symmetry, eMChE can generally appear in chiral systems. Resistivities with current density **j** parallel and antiparallel to magnetic field **B** exhibit different values^5–7^. Resistivity considering eMChE can be described as follows:

1 $${\bf{E}} = \rho \left[ {1 + {{\hat \gamma }^{{\rm{R}}/{\rm{L}}}}\left( B \right)\left( {{\bf{j}} \cdot {\bf{B}}} \right)} \right]{\bf{j}}.$$

Here, *ρ* is the linear term of longitudinal resistivity, R and L denote right- and left-handed crystalline chiralities, and ${\hat \gamma ^{{\rm{R}}/{\rm{L}}}}$ (*B*) is the eMChE coefficient being an even function of *B*. We schematize the current-directional response in MnSi for each experimental configuration in Fig. 1d. Note that Eq. (1) can be transformed to the equivalent form, ${\bf{j}} = \left( {\frac{1}{\rho }} \right)\left[ {1 - {{\hat \gamma }^{{\rm{R}}/{\rm{L}}}}\left( B \right)\left( {{\bf{E}} \cdot {\bf{B}}} \right){\rm{/}}{\rho ^2}} \right]{\bf{E}}$. Under time-reversal operation, the current direction for higher conductance is reversed. Likewise, the higher-conductance direction is opposite for different crystal chiralities; ${\hat \gamma ^{\rm{R}}}(B) = - {\hat \gamma ^{\rm{L}}}(B)$. Since voltage signals from eMChE are anticipated to be small, enough large current density is required to detect eMChE. In order to increase current density under the limitation of external high-precision current sources, by using focused ion beam (FIB) we fabricated microscale thin plates of MnSi, whose thickness and width are approximately 500 nm and 10 μm, respectively (Fig. 1e). Temperature dependence of resistivities and magnetic phase diagram of thin plate samples resemble those of bulk samples (see Supplementary Fig. 1 and Supplementary Note 1).

### Electrical magnetochiral effect at ambient pressure

**Fig. 2:**
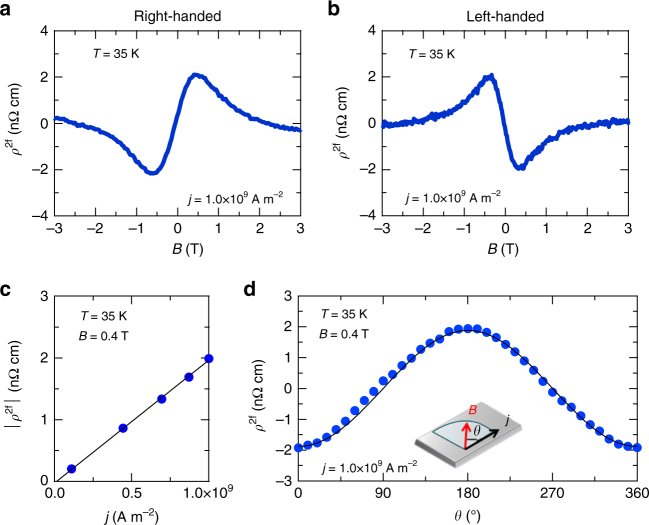
Electrical magnetochiral effect in MnSi thin plate samples. **a**, **b** Magnetic field dependence of second harmonic resistivity (*ρ* ^2f^) in right-handed **a** and left-handed MnSi crystals **b**. **c**, **d** Current-density (*j*) dependence of *ρ* ^2f^ **c** and angle (*θ*) dependence of *ρ* ^2f^ **d** in left-handed MnSi. Here *θ* is the angle between current and the magnetic field as shown in the inset of **d**. The solid line is fit to cos*θ*

First, we show typical profiles of eMChE signals observed in MnSi. Since eMChE appears as a nonlinear transport response in proportion to *j* ^2^ (Eq. (1)), we measured second harmonic resistivity (*ρ* ^2f^), which is directly connected to eMChE as ${\rho ^{2{\rm{f}}}} = \frac{\rho }{2}{\hat \gamma ^{{\rm{R}}/{\rm{L}}}}\left( B \right)\left( {{\bf{j}} \cdot {\bf{B}}} \right)$ (see Supplementary Note 2). The magnetic field and current were applied parallel to [100] direction unless otherwise noted. Figure 2a, b present *ρ* ^2f^ of right- and left-handed MnSi at *T* = 35 K with current density *j* = 1.0 × 10^9^ Am^−2^ and frequency *f* = 30.5 Hz. The both right- and left-handed crystals were selected from several batches by identifying the handedness in terms of the conversion beam electron diffraction method (see Supplementary Fig. 2 and Supplementary Note 3). In accord with the expected contributions from eMChE, both the field profiles of *ρ* ^2f^ of the right- and left-handed crystals are antisymmetric against **B**, exhibiting the opposite sign to each other. To further confirm that the observed *ρ* ^2f^ signals stem from eMChE, we measured *ρ* ^2f^ at *B* = 0.4 T as functions of current density and relative angle *θ* between **B** and **j** both lying in-plane (Fig. 2c, d). Both the *j*- and *θ*-dependences obey the expected behaviours from the relation ${\rho ^{2{\rm{f}}}} = \frac{\rho }{2}{\hat \gamma ^{{\rm{R}}/{\rm{L}}}}\left( B \right)\left( {{\bf{j}} \cdot {\bf{B}}} \right)$; *ρ* ^2f^ is proportional to *j* and cos*θ*, respectively. We evaluate ${\hat \gamma ^{{\rm{R}}/{\rm{L}}}}$ from the fitting of the angular dependence of *ρ* ^2f^ by the equation ${\rho ^{2{\rm{f}}}} = \frac{\rho }{2}{\hat \gamma ^{{\rm{R}}/{\rm{L}}}}\left( B \right)\left( {{\bf{j}} \cdot {\bf{B}}} \right)$ (see also the solid line of Fig. 2d for the fitting curve). The magnitude of ${\hat \gamma ^{{\rm{R}}/{\rm{L}}}}(B)$ at *B* = 0.4 T is 1.8 × 10^−13^ m^2^T^−1^A^−1^, lying within a range of ${\hat \gamma ^{{\rm{R}}/{\rm{L}}}}\left( B \right)$ values reported for non-magnetic chiral materials (~10^−8^–10^−14^ m^2^T^−1^A^−1^)^7^. We note that the observed second harmonic resistivity does not result from Nernst effect, which is proposed as a possible origin of second harmonic resistivity^8, 10^. While Nernst voltage might be, more or less, generated perpendicular to the magnetic field, the observed angular dependence of *ρ* ^2f^ indicates that the second harmonic voltage is produced parallel to the magnetic field.

**Fig. 3:**
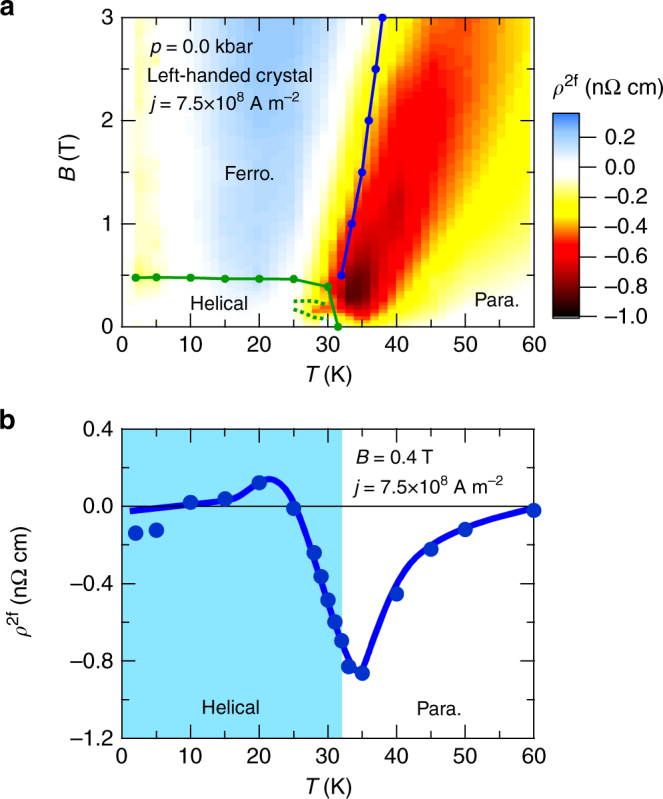
Temperature dependence of electrical magnetochiral effect. **a** Contour mapping of second harmonic resistivity (*ρ* ^2f^) in left-handed MnSi in *T*-*B* plane. The green and blue lines denote the phase boundary enclosing the helical phase and the crossover line between the induced ferromagnetic and paramagnetic phases, respectively. For the *ρ* ^2f^ anomaly around the narrow skyrmion-lattice phase region (denoted by a dotted green line), see Supplementary Fig. 4 and Supplementary Note 5. **b** Temperature dependence of *ρ* ^2f^ at *B* = 0.4 T

Next, we discuss a dominant mechanism of eMChE in MnSi. One mechanism proposed for eMChE in non-magnetic materials is so-called self-field effect^5^. In this mechanism, eMChE is expected to show *B*-linear dependence. This is however inconsistent with the present observation that *ρ* ^2f^ is suppressed at high magnetic field as presented in Fig. 2a, b. Another possible mechanism of eMChE is asymmetric electron scatterings by chiral scatterers^5^. To examine this, we investigate *T*- and *B*-dependences of *ρ* ^2f^. In Fig. 3a, we show a contour mapping of *ρ* ^2f^ in the *T*-*B* plane for left-handed MnSi, measured with *j* = 7.5 × 10^8^ Am^−2^. We determined the helical-to-ferromagnetic phase boundary and ferromagnetic-to-paramagnetic crossover line from kinks in *B*-dependence of the planar Hall resistivity^19^ and inflection points of *ρ* − *T* curve, respectively. Second harmonic resistivity becomes prominent in the paramagnetic region, showing the broad peak profile in the *T*-*B* plane just above the phase boundary (helical-to-paramagnetic) and the crossover line (ferromagnetic-to-paramagnetic). In contrast, the signal suddenly declines with entering the long-range ordering phases. These behaviours are exemplified by the *T*-scan of *ρ* ^2f^ at *B* = 0.4 T as shown in Fig. 3b; the magnitude of *ρ* ^2f^ exhibits its maximum near *T*_c_, and shows sharper decrease at the side of helical phase than at the side of paramagnetic phase. Here, we defined the transition temperature for the helical ordering as the temperature where *ρ* − *T* curve shows an inflection (see Supplementary Fig. 1 and Supplementary Note 1). We note that *ρ* ^2f^ for right-handed MnSi qualitatively shows similar *T*- and *B*-dependences to *ρ* ^2f^ for left-handed MnSi, apart from the reversed sign (see Supplementary Fig. 3 for a contour mapping of *ρ* ^2f^ for right-handed MnSi and Supplementary Note 4). The above results indicate that eMChE in MnSi is related to the strongly enhanced chiral spin fluctuations around and immediately above *T*_c_^14–17^, which should induce asymmetric electron scatterings. This scattering process of spin-polarized conduction electrons may share the common microscopic mechanism with asymmetric scatterings of polarized neutrons by chiral spin fluctuations^14–17^. It is worth noting here that eMChE is also observed at the phase boundary between the conical and skyrmion-lattice states (see Supplementary Fig. 4 and Supplementary Note 5).

### Electrical magnetochiral effect in partial order phase

**Fig. 4:**
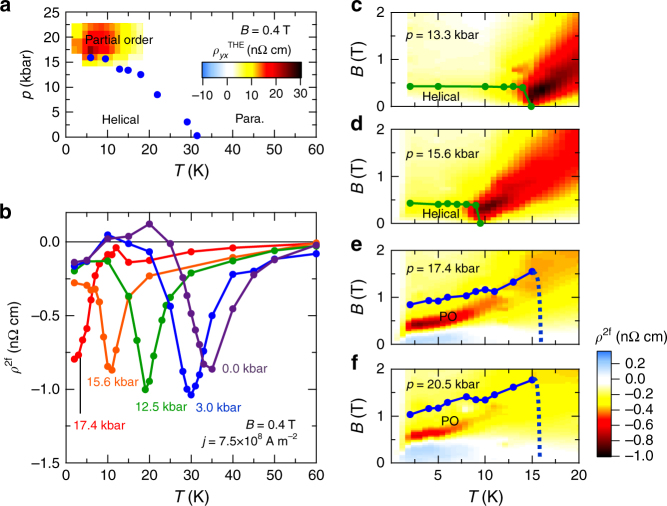
Pressure effect on electrical magnetochiral effect. **a** Pressure (*p*)—Temperature (*T*) phase diagram together with contour mapping of observed topological Hall resistivity $\rho _{yx}^{{\rm{THE}}}$ at 0.4 T (see Supplementary Fig. 5 and Supplementary Note 6). **b** Temperature dependence of second harmonic resistivity (*ρ* ^2f^) for 0.4 T at various pressures in left-handed MnSi. **c**–**f** Contour mappings of *ρ* ^2f^ at various pressures in *T*- *B* plane in left-handed MnSi. The green lines are the phase boundary between the conical and induced ferromagnetic phases determined from magnetoresistivity measurements, and the blue lines are the phase boundary of the partial order phase determined from topological Hall effect measurements

Up to this point, we have revealed that the eMChE in MnSi arises from thermal spin fluctuations enhanced in the vicinity of the helical order as well as of the skyrmion-lattice phase. Next, we investigate the possible effect of quantum spin fluctuations on eMChE. In bulk samples of MnSi, the long-range static helical order is suppressed under pressure, and disappears at a pressure of *p* = 14.6 kbar^20–22^, where the quantum phase transition occurs and consequently the quantum spin fluctuations become dominant. Even above the pressure for this quantum phase transition, there exists a dynamical topological magnetic order, which fluctuates on time scales between 10^−10^ and 10^−11^ s^20–22^. Since this dynamical magnetic order, called partial order (PO), is promoted by quantum fluctuations, the investigation of eMChE in the PO state will provide us with insight into effects of quantum chiral spin fluctuations. In Fig. 4a, we show *p*-*T* phase diagram determined from *T*-dependence of resistivity and topological Hall resistivity $\rho _{yx}^{{\rm{THE}}}$ (for the experimental details, see Supplementary Fig. 5 and Supplementary Note 6). The emergence of $\rho _{yx}^{{\rm{THE}}}$ in the PO state is a hallmark of the topological spin correlation endowed with the scalar spin chirality^22^. The *p*-*T* phase diagram is almost identical to that of bulk sample except for increase of the critical pressure *p*_c_ (~17 kbar). The increased *p*_c_ is probably due to the tensile strain from the Si sample stage, which compensates the effect of applied hydrostatic pressure.

Figure 4b shows *T*-dependence of *ρ* ^2f^ in a left-handed MnSi thin plate sample under *B* = 0.4 T at various pressures, measured with *j* = 7.5 × 10^8^ Am^−2^. Contour mappings of *ρ* ^2f^ in *T*-*B* plane are presented at several pressures in Fig. 4c–f. Here, we determined the helical-to-ferromagnetic and the partial order-to-ferromagnetic phase boundaries from the inflection points of *ρ* − *B* curves and magnetic field where $\rho _{yx}^{{\rm{THE}}}$ disappeared, respectively. For *p* < *p*_c_ ≈ 17 kbar, a large magnitude of eMChE signal is detected at the periphery just above *T*_c_, like the case under the ambient pressure, as seen from Fig. 4a–d (see also Fig. 3a, b for *ρ* ^2f^ at *p* = 0 kbar). For *p* > *p*_c_ ≈ 17 kbar, the eMChE signal shows the maximum magnitude at the lowest measurement temperature within the PO phase, not around the boundaries between the PO and ferromagnetic states nor between the PO and paramagnetic states (Fig. 4e, f). This feature suggests that the eMChE under *p* > *p*_c_ is induced by the quantum spin fluctuations or dynamics of the PO state, which should possess the chiral nature as well.

Lastly, we comment on the existence of eMChE signals with positive (negative) sign in the left-handed (right-handed) MnSi, which show the opposite sign to those induced by thermal and quantum spin fluctuations as discussed above. The positive *ρ* ^2f^ signals in the left-handed crystal are observed at portions of helical and ferromagnetic phases at ambient pressure (blue region of Fig. 3a) and at low magnetic fields in the PO phase (blue regions of Fig. 4e, f). Magnetic-field scans in the temperature region with positive *ρ* ^2f^ at ambient pressure (*T* = 10–25 K) indicates that the eMChE signals vary in proportion to magnetization (see Supplementary Fig. 6 and Supplementary Note 7). This implies contribution from another mechanism for eMChE related not to the spin fluctuations but to the static magnetization. Observation of such a different sign of *ρ* ^2f^ in the low-field region of the PO phase may also capture some static or frozen-order nature of the PO state.

## Discussion

In conclusion, thermal and quantum spin fluctuations in the chiral magnet MnSi, which are critically enhanced in association with classical and quantum phase transitions, give rise to asymmetric electron scatterings, leading to the large eMChE. Our finding sheds light not only on the cooperative phenomenon from spin fluctuations and spin chirality but also on the novel functionality in chiral magnets^23^, such as the directional nonlinear magnetotransport.

## Methods

### Sample preparation

Single crystals of MnSi were synthesized with use of the Czochralski method. Their crystalline chirality was confirmed by using convergent beam electron diffraction (CBED) method (see also Supplementary Fig. 2 and Supplementary Note 3). By focused ion beam (FIB) technique (NB-5000, Hitachi), we cut the thin plates out of those single crystals. Sizes of the thin plates are typically ∼10 μm × 20 μm × 500 nm. The thin plates were mounted on a silicon stage and were fixed with the use of FIB-assisted tungsten-deposition. Gold electrodes were patterned by using photolithography and electron beam deposition. We prepared several thin-plate samples to confirm the reproducibility.

### Transport measurements

Linear longitudinal resistivity and Hall resistivity were measured by using dc-transport option of Physical Property Measurement System (PPMS). Hydrostatic pressures were applied with use of a CuBe clamp cell, and the applied pressures were calibrated with pressure change of superconducting transition temperature of Pb. Second harmonic resistivity was measured by using a Lock-in technique (SR-830, Stanford Research Systems); we input low-frequency (*f*) ac current and measured second harmonic resistivity.

### Data availability

The data that support the findings of this study are available from the corresponding author upon reasonable request.

## Electronic supplementary material

Supplementary Information
