# Supplementary material for: Electrical magnetochiral effect induced by chiral spin fluctuations
Source: Nat Commun. 2017 Oct 11;8:866. doi: 10.1038/s41467-017-01094-2 (PMC5636803; doi:10.1038/s41467-017-01094-2)
Supplement: Supplementary file 1 — Supplementary Information [file 41467_2017_1094_MOESM1_ESM.pdf]

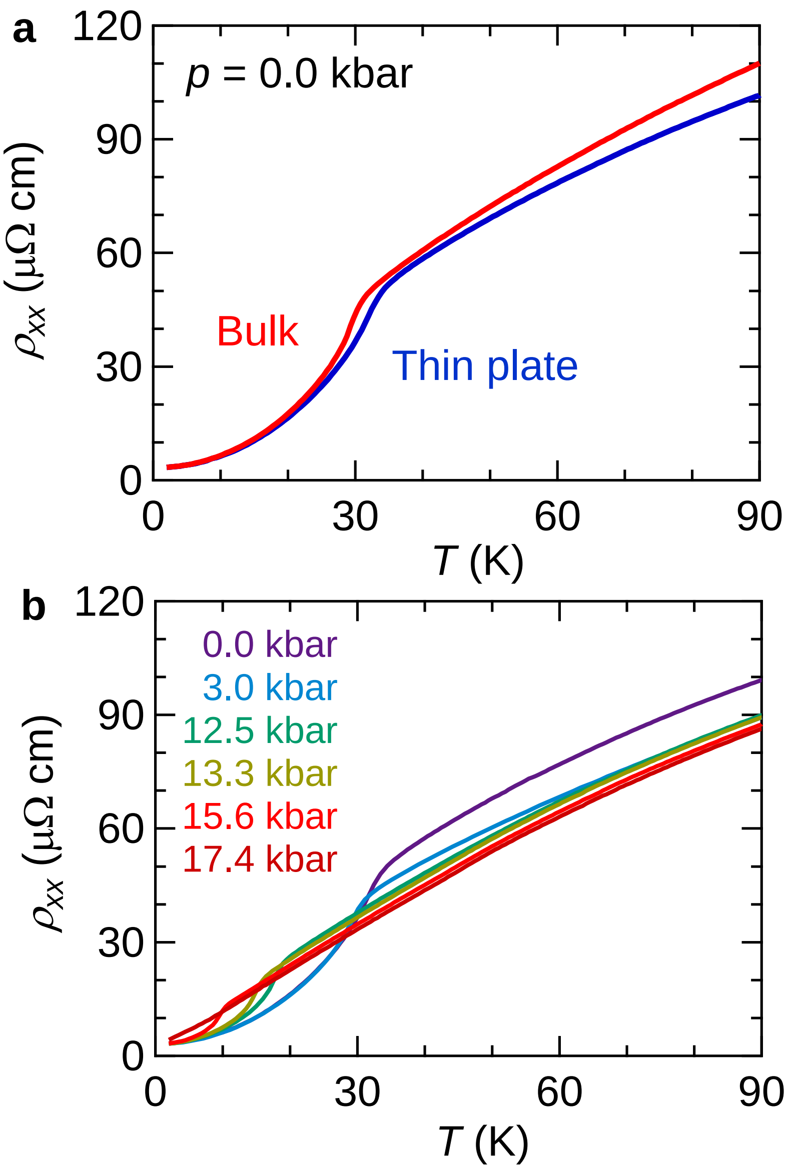

Supplementary Figure 1 │Temperature dependence of longitudinal resistivity. a, Comparison of temperature (T) dependence of resistivity (ρ_{xx}) between bulk sample (red line) and thin plate sample of MnSi (blue line). Resistivities of two samples show similar T-dependence, indicating minimal damage due to fabrication process by focused ion beam (FIB) technique. **b**, Resistivity of thin plate sample at various pressures. The magnetic transition temperatures are determined from the inflection points of ρ_{xx}-T curves. The transition temperatures decrease with increasing applied pressure, resulting in the disappearance of the helical transition above p = 17.4 kbar.

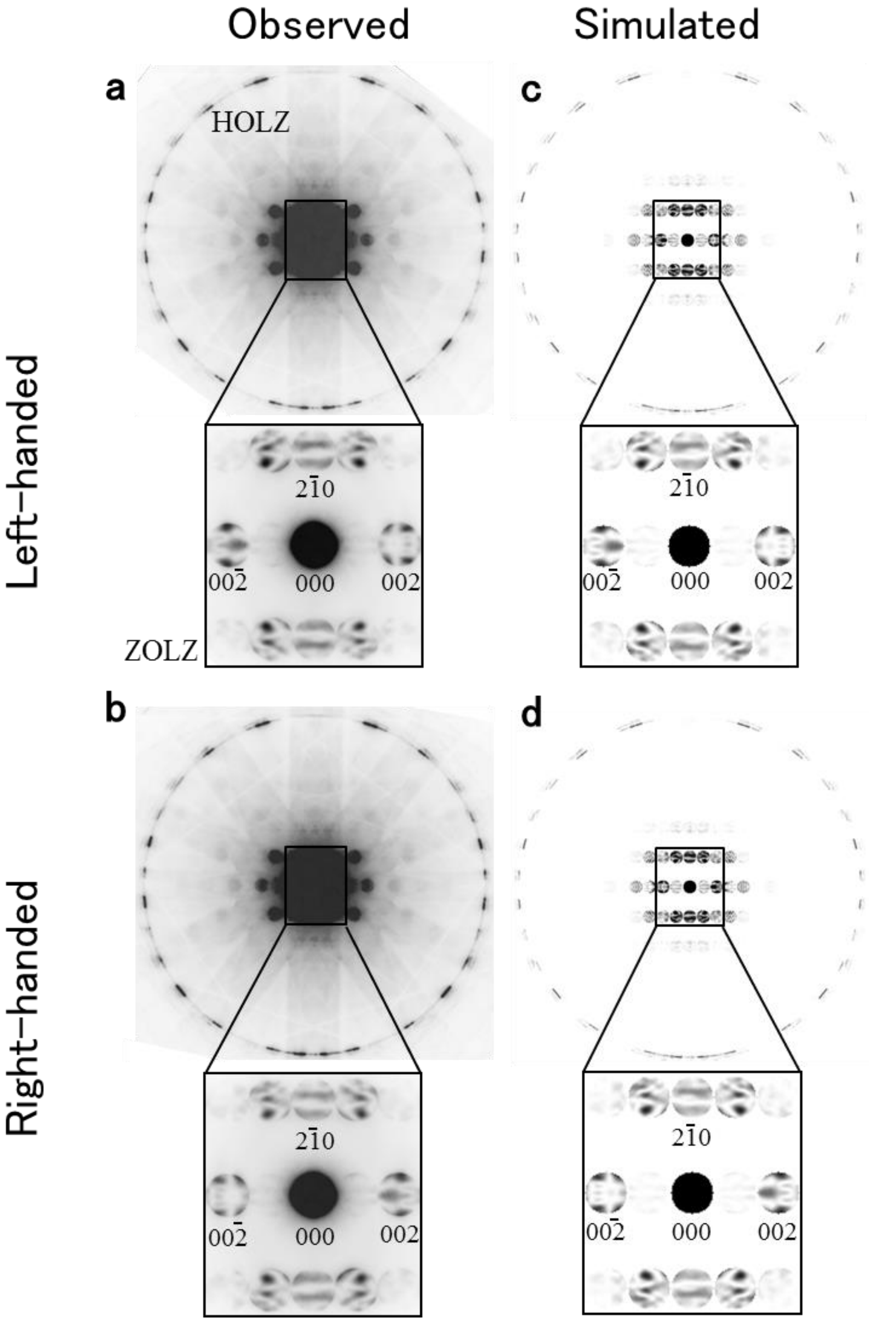

Supplementary Figure 2 │Convergent beam electron diffraction patterns (CBED). a, **b**, Observed CBED patterns taken with [120] incidence from different single crystals. Those patterns are matched to simulated CBED patterns assuming (**c**) the left- and (**d**) right-handed crystal structures, respectively.

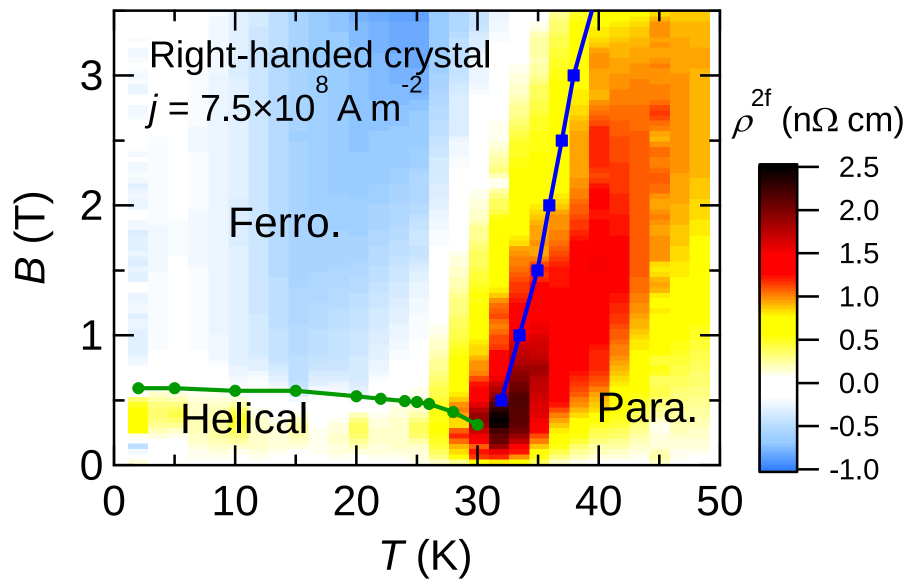

Supplementary Figure 3 | Contour mapping of second harmonic resistivity in right-handed MnSi. Contour mapping of second harmonic resistivity (ρ^{2f}) in right-handed MnSi in *T*-*B* plane, measured with $j = 7.5 \times 10^{8}$ A m^{-2}. Note that the sign of ρ^{2f} is reversed as compared with the case of left-handed MnSi (Fig. 3**a**). The green and blue lines denote the phase boundary enclosing the helical phase and the crossover line between the induced ferromagnetic and paramagnetic phases, respectively.

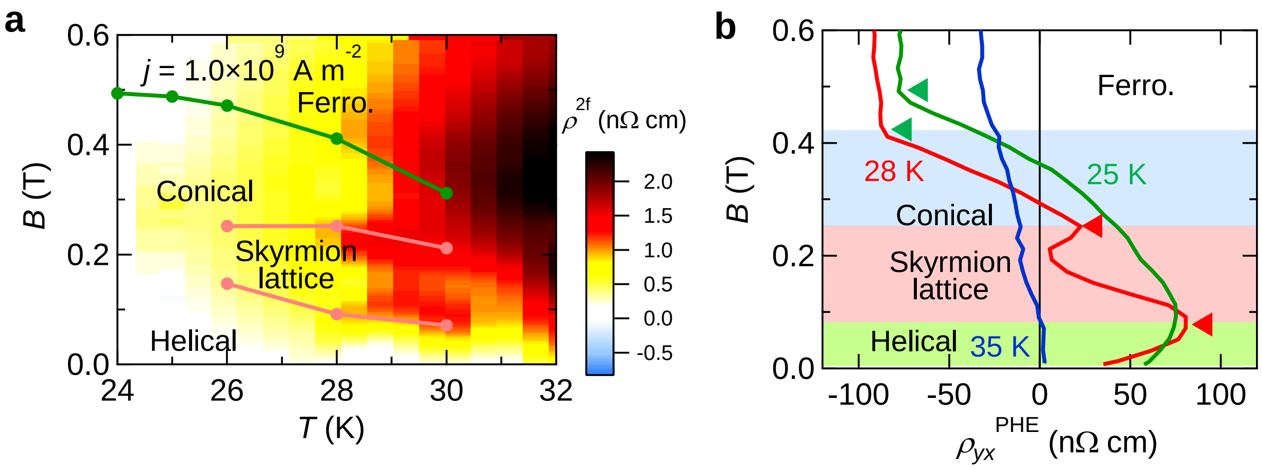

Supplementary Figure 4 │Electrical magnetochiral effect at the phase boundary of the skyrmion-lattice states. a, Contour mapping of ρ^{2f} of right-handed MnSi around skyrmion phase. **b,** Magnetic field dependence of planar Hall resistivity ρ^{PHE} at various temperatures. The green and red triangles represent the phase transitions between the ferromagnetic and conical phases and between the conical and skyrmion-lattice phases, respectively.

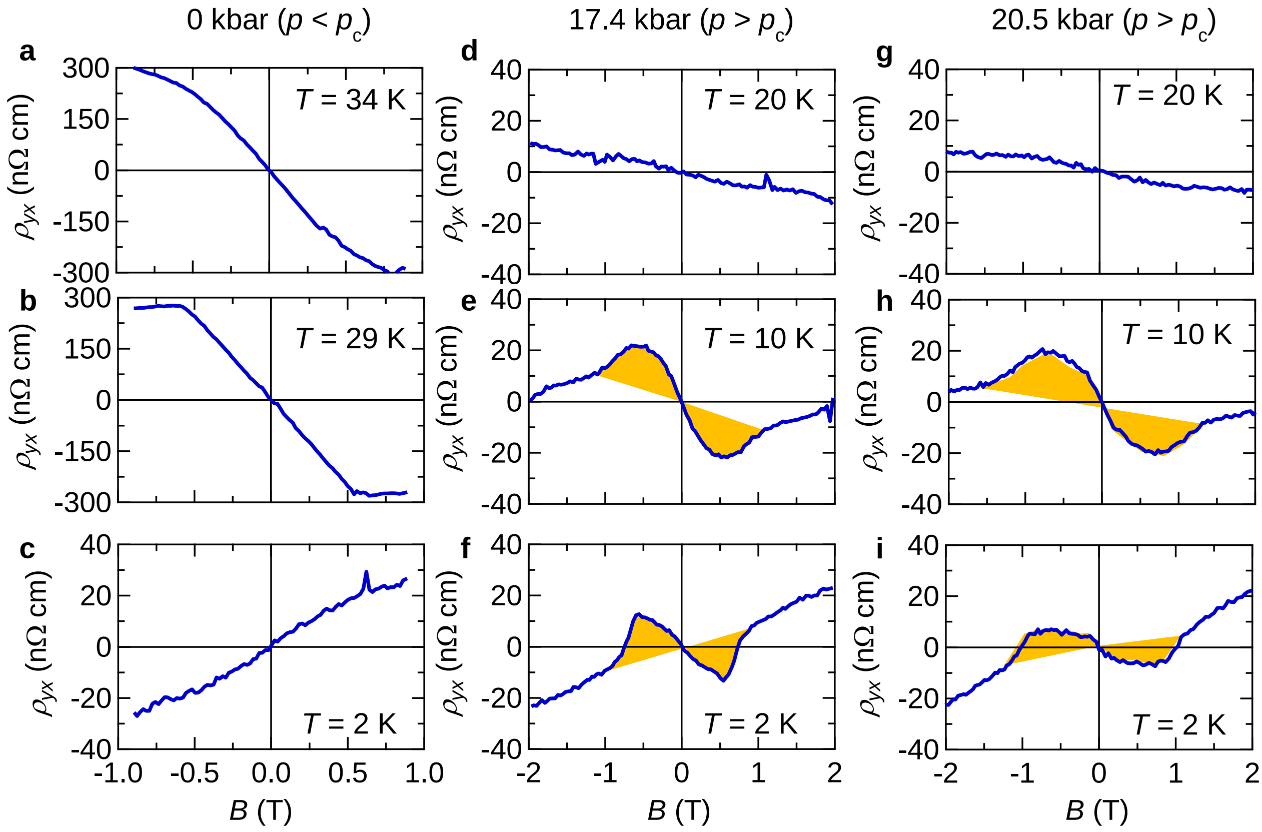

Supplementary Figure 5 │Magnetic-field dependence of Hall resistivity under various pressures. a-c, Magnetic-field dependence of Hall resistivity (ρ_{yx}) at various temperatures under p = 0 kbar. **d-i**, Magnetic-field dependence of ρ_{yx} at various temperatures under 17.4 kbar (**d-f**) and 20.5 kbar (**g-i**) above the critical pressure (p_c), where long-range helical ordering is suppressed. The orange shadows represent contribution of topological Hall effect. The distinctive topological Hall signals verify the formation of a topological spin structure above p_c in the thin-plate samples of MnSi.

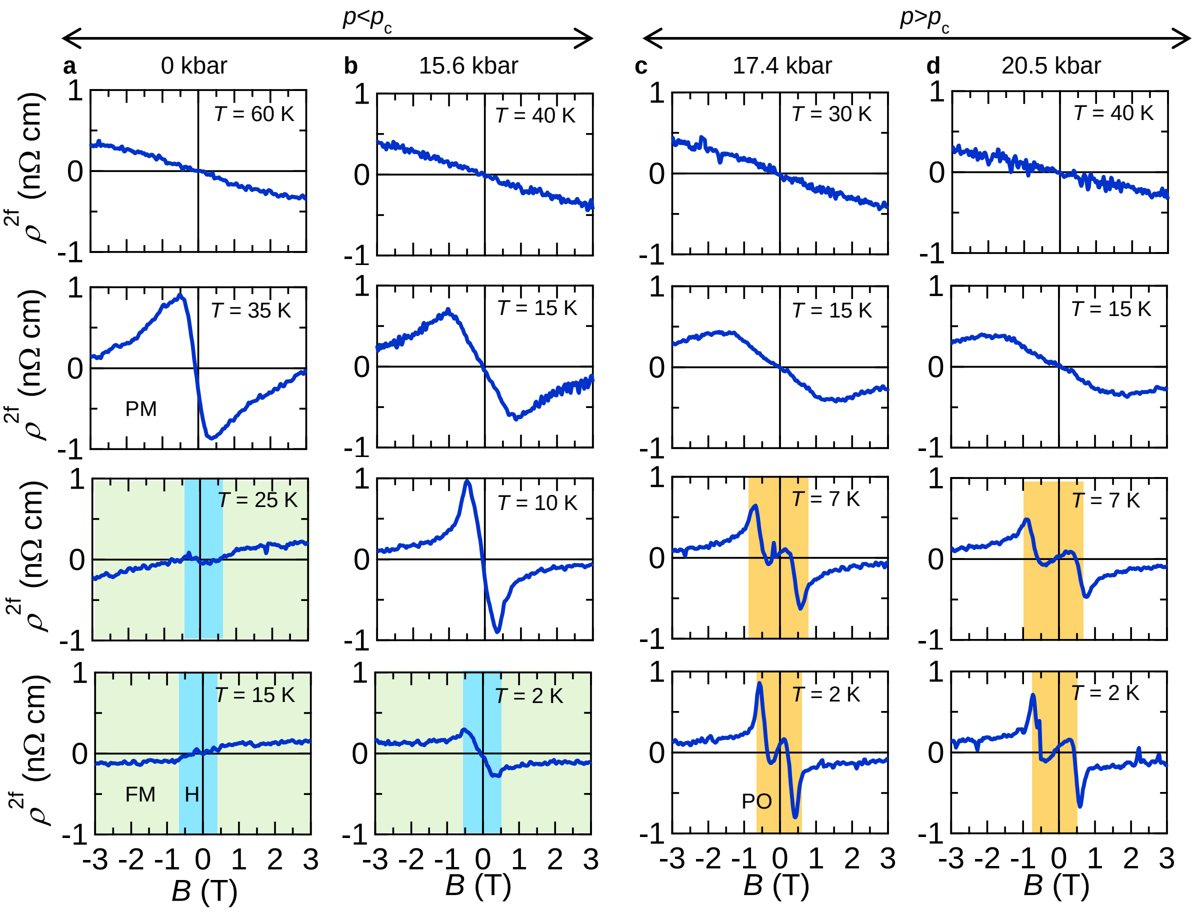

Supplementary Figure 6 │Magnetic-field dependence of electrical magnetochiral effect under various pressures. a, b, Magnetic-field dependence of second harmonic resistivity (ρ^{2f}) below the critical pressure (p_c). The blue and green shadows represent the helical (H) and ferromagnetic (FM) phases, respectively. **c, d,** Magnetic-field dpendence of ρ^{2f} above p_c. The orange shadows represent the partial order (PO) phase.

Supplementary Note 1 │Temperature dependence of resistivity in MnSi thin plate samples.

In Supplementary Figure 1**a**, we compare linear longitudinal resistivity (ρ_{xx}) of a MnSi thin plate sample with that of bulk single crystal, out of which we sliced the thin plate. Resistivities of two samples show similar temperature (T) dependence, indicating minimal damage due to FIB fabrication process. We determined the transition temperature of the helical ordering as the temperature where ρ_{xx}-T curve exhibits an inflection. Note that the slight increase of transition temperature in the thin plate sample compared to that of the bulk is due to perhaps uniaxial strains from the silicon sample stage[1].

In Supplementary Figure 1**b**, we present T-dependence of resistivity of the thin plate sample at various pressures. We also assigned inflection points of ρ_{xx}-T curves to the magnetic transition temperatures. The transition temperatures decrease with increasing applied pressure, and above p = 17.4 kbar, the helical transition disappears.

Supplementary Note 2 │Second harmonic resistivity.

The second harmonic resistivity is defined as

$$\rho^{2\mathrm{f}} = \frac{V^{2\mathrm{f}} S}{I\, d} = \frac{V^{2\mathrm{f}}}{j\, d} \quad (1).$$

Here, $V^{2\mathrm{f}}$, I, S, j, and d are second harmonic voltage, current, cross-sectional area of the MnSi thin plate, current density, and distance between voltage terminals, respectively. The relation between $\rho^{2\mathrm{f}}$ and electrical magnetochiral effect (eMChE) is derived as follows. When we input ac current $\hat{\mathbf{j}} = \mathbf{j}\sin\omega t$, the electrical magnetochiral voltage V^{eMChE} is expressed as

$$V^{\mathrm{eMChE}} = \rho\left[\,\hat{\gamma}^{\mathrm{R/L}}(\hat{\mathbf{j}}\cdot\mathbf{B})\right]\hat{j}d = \rho\left[\,\hat{\gamma}^{\mathrm{R/L}}(\mathbf{j}\cdot\mathbf{B})\right]jd\,(\sin\omega t)^2$$

$$= \frac{\rho}{2}\left[\,\hat{\gamma}^{\mathrm{R/L}}(\mathbf{j}\cdot\mathbf{B})\right]jd[1+\cos(2\omega t)]. \quad (2)$$

Here the coefficient of $\cos(2\omega t)$ is second harmonic voltage, and therefore we obtain

$$V^{2\mathrm{f}} = \frac{\rho}{2}\left[\hat{\gamma}^{\mathrm{R/L}}(\mathbf{j}\cdot\mathbf{B})\right]jd. \qquad (3)$$

From supplementary equations (1) and (3), $V^{2\mathrm{f}}$ is related to eMChE as

$$\rho^{2\mathrm{f}} = \frac{V^{2\mathrm{f}}}{j\,d} = \frac{\rho}{2}\hat{\gamma}^{\mathrm{R/L}}(\mathbf{j}\cdot\mathbf{B}). \qquad (4)$$

Supplementary Note 3 │Convergent beam electron diffraction.

Crystalline chirality of MnSi was confirmed by using convergent beam electron diffraction (CBED) method[2]. Supplementary Figure 2 shows the observed CBED patterns of the left- and right-handed MnSi crystals in comparison with the simulated ones by using software MBFIT[2].

Supplementary Note 4 │Temperature and magnetic field dependences of second-harmonic resistivity in right-handed MnSi.

A contour mapping of $\rho^{2\mathrm{f}}$ in the *T*-*B* plan for right-handed MnSi is shown in Supplementary Figure 3. The *T*- and *B*- dependences for right-handed MnSi are qualitatively similar to those for left-handed MnSi, except for the sign reversal. There observed a small difference in the maximum value of $\rho^{2\mathrm{f}}$ between right-handed MnSi ($\rho^{2\mathrm{f}}\sim$ 3 nΩ cm) and left- handed MnSi ($\rho^{2\mathrm{f}}\sim$ 1 nΩ cm), which may be due to different sample quality such as population of magnetic pinning centres suppressing spin fluctuations.

Supplementary Note 5 │Electrical magnetochiral effect at the phase boundary of the skyrmion-lattice states.

Electrical magnetochiral effect is also observed at the phase boundary between the conical and skyrmion-lattice states. To precisely estimate the phase boundary, we employed

measurements of planar Hall effect (PHE), which was proven in the former study[3] to sensitively detect variations in anisotropic magnetoresistance associated with magnetic transitions, typically showing kinks at phase boundaries between skyrmion-lattice, conical, and induced ferromagnetic states. In Supplementary Figure 4**b**, we present *B*-dependence of planar Hall resistivity (ρ^{PHE}) around T_{c}, marking the phase boundaries as red and green triangles. The magnetic phase diagram for the thin plate sample is also similar to that for bulk crystal, except for slight expansion of the skyrmion-lattice phase region. Incidentally, the stabilization of skyrmion state are attributed to uniaxial strain, which arises from difference in thermal expansion between the MnSi thin plate and the sample stage made of Si (Ref. 4) A contour mapping of $\rho^{2\mathrm{f}}$ in a right-handed MnSi sample measured with $j = 1.0 \times 10^9\ \mathrm{Am}^{-2}$ is presented in Supplementary Figure 4**a**, together with the phase boundaries determined from these ρ^{PHE}-mesurements. This clearly captures the enhanced magnitude of $\rho^{2\mathrm{f}}$ at the boundaries of skyrmion-lattice phase, indicating that the asymmetric electron scattering by the chiral spin fluctuations also manifests itself at the phase transition between the skyrmion-lattice and the conical or helical states.

Supplementary Note 6 | Topological Hall effect in MnSi under pressure.

In conventional magnets, ordinary Hall effect and anomalous Hall effect contribute to Hall resistivity as $\rho_{yx} = R_0 B + R_{\mathrm{S}} M$. Here, $B, M, R_0,$ and R_{S} are magnetic field, magnetization, ordinal Hall coefficient, and anomalous Hall coefficient, respectively. In a noncoplanar spin structure with nonzero topological winding number, such as skyrmions, an additional Hall effect called topological Hall effect shows up[5]. In the case of MnSi, the topological Hall effect is observed not only in the narrow temperature (*T*)-magnetic field (*B*) region of skyrmion phase, but also in a wide *T*-*B* region of the partial order (PO) phase, where applied pressure exceeds the critical pressure for the disappearance of helical order (p_{c}). This indicates the existence of a topological spin structure above p_{c} (Ref. 6). We measured the Hall resistivity of MnSi thin plates and reproduced the similar signals of topological Hall

effect as reported in MnSi bulk sample. In Supplementary Figure 5, we show magnetic-field dependence of Hall resistivity in a MnSi thin plate sample at various temperatures and pressures, in accord with the results reported in Ref. 6. Hall resistivity at p = 0 kbar is dominated by sum of ordinary and anomalous Hall signals (Supplementary Figure 5**a** – **c**). In contrast, above p_c, we found the additional contribution of topological Hall effect as indicated by orange shadows (Supplementary Figure 5**d** – **i**). The observation of topological Hall effect identifies the existence of topological spin structure above p_c even in thin plate samples of MnSi.

Supplementary Note 7 │Magnetic-field dependence of electrical magnetochiral effect at various temperatures and pressures.

In Supplementary Figure 6, we show magnetic-field dependence of ρ^{2f} in a left-handed MnSi measured with current density $j = 7.5 \times 10^8$ Am^{-2} at various temperatures and pressures. The helical phases, ferromagnetic phases, and partial magnetic order phases are highlighted by light blue, green, and orange shadows, respectively.

Supplementary References

1. Li, Yufan, Kanazawa, N., Yu, X. Z., Tsukazaki, A., Kawasaki, M., Ichikawa, M., Jin, X. F., Kagawa, F. & Tokura, Y. Robust Formation of Skyrmions and Topological Hall Effect Anomaly in Epitaxial Thin Films of MnSi. *Phys. Rev. Lett*. **110**, 117202 (2013).

2. Tsuda, K., & Tanaka, M., Refinement of crystal structural parameters using two-dimensional energy-filtered CBED patterns. *Acta Cryst*. **A 55**, 939 (1999).

3. Yokouchi, T., Kanazawa, N., Tsukazaki, A., Kozuka, Y., Kikkawa, A., Taguchi, Y., Kawasaki, M., Ichikawa, M., Kagawa, F., & Tokura, Y., Formation of In-plane

Skyrmions in Epitaxial MnSi Thin Films as Revealed by Planar Hall Effect. *J. Phys. Soc. Jpn.* **84**, 104708 (2015).

4. Nii, Y., Nakajima, T., Kikkawa, A., Yamasaki, Y., Ohishi, K., Suzuki, J., Taguchi, Y. Arima, T., Tokura, Y. & Iwasa, Y., Uniaxial stress control of skyrmion phase. *Nat. Commun.* **6,** 8539 (2015).

5. Bruno, P., Dugaev, V. K. & Taillefumier, M., Topological Hall Effect and Berry Phase in Magnetic Nanostructures. *Phys. Rev. Lett.* **93**, 096806 (2004).

6. Ritz, R., Halder, M., Wagner, M., Franz, C., Bauer, A. & Pfleiderer, C. Formation of a topological non-Fermi liquid in MnSi. *Nature* **497**, 231 (2013).
